# Investigation of the Causes of Shigatoxigenic *Escherichia coli* PCR Positive and Culture Negative Samples

**DOI:** 10.3390/microorganisms8040587

**Published:** 2020-04-18

**Authors:** Guerrino Macori, Siobhán C. McCarthy, Catherine M. Burgess, Séamus Fanning, Geraldine Duffy

**Affiliations:** 1 Teagasc Food Research Centre, Ashtown, Dublin 15, Ireland; siobhan.c.mccarthy@teagasc.ie (S.C.M.); Geraldine.Duffy@teagasc.ie (G.D.); 2 UCD-Centre for Food Safety, School of Public Health, Physiotherapy & Sports Science, University College Dublin, Belfield, Dublin D04 N2E5, Ireland; sfanning@ucd.ie

**Keywords:** STEC, qPCR, bacteriophages, Stx phages, PMA, VBNC

## Abstract

Molecular methods may reveal the presence of pathogens in samples through the detection of specific target gene(s) associated with microorganisms, but often, the subsequent cultural isolation of the pathogen is not possible. This discrepancy may be related to low concentration of the cells, presence of dead cells, competitive microflora, injured cells and cells in a viable but non-culturable state, free DNA and the presence of free bacteriophages which can carry the target gene causing the PCR-positive/culture-negative results. Shiga-toxigenic *Escherichia coli* (STEC) was used as a model for studying this phenomenon, based on the phage-encoded cytotoxins genes (Stx family) as the detection target in samples through real-time qPCR. Stx phages can be integrated in the STEC chromosome or can be isolated as free particles in the environment. In this study, a combination of PCR with culturing was used for investigating the presence of the *stx1* and *stx2* genes in 155 ovine recto-anal junction swab samples (method (a)-PCR). Samples which were PCR-positive and culture-negative were subjected to additional analyses including detection of dead STEC cells (method (b)-PCR-PMA dye assay), presence of Stx phages (method (c)-plaque assays) and inducible integrated phages (method (d)-phage induction). Method (a) showed that even though 121 samples gave a PCR-positive result (78%), only 68 samples yielded a culturable isolate (43.9%). Among the 53 (34.2%) PCR-positive/culture-negative samples, 21 (39.6%) samples were shown to have STEC dead cells only, eight (15.1%) had a combination of dead cells and inducible *stx* phage, while two samples (3.8%) had a combination of dead cells, inducible phage and free *stx* phage, and a further two samples had Stx1 free phages only (3.8%). It was thus possible to reduce the samples with no explanation to 20 (37.7% of 53 samples), representing a further step towards an improved understanding of the STEC PCR-positive/culture-negative phenomenon.

## 1. Introduction

Shigatoxigenic *Escherichia coli* (STEC) are major zoonotic foodborne pathogens capable of causing severe gastrointestinal diseases and complications such as haemorrhagic colitis (HC) and haemolytic uremic syndrome (HUS) [1,2]. The main virulence factors of STEC are the phage-encoded cytotoxins called Shiga toxins (Stx family), with several toxin subtypes [3]. Stx phages can be present, either integrated in the STEC chromosome as temperate bacteriophage or as free particles in the environment [4,5,6]. STEC strains are genetically heterogeneous and grouped as serotypes, and the serotype O157:H7 has been reported as the causative agent of large outbreaks of food-borne disease and most reported in the literature [7,8,9,10]. However, the emergence of non-O157 serotypes as agents responsible for foodborne outbreaks including O26:H11, O111:NM, O103:H2, and O145:NM with different combinations of toxin subtypes [11,12,13,14,15,16,17], has increased the importance of developing effective methods for the detection and characterization of a wider range of STEC [18]. Culture-dependent detection methods for screening STEC in samples include enrichment in selective media and subsequent use of DNA-based methods for their detection and quantification such as real-time quantitative PCR (qPCR) for *stx* genes, followed by cultural isolation (ISO, 2012; CEC, 2005-EC 2073).

Even though PCR- and qPCR-positive results may indicate the presence of *stx* in a sample, often, the subsequent cultural isolation of the STEC strain is not possible, with large discrepancies reported between PCR- and culture-positive results across a range of sample matrices. This is a common finding in PCR-based STEC detection, in ovine [19], bovine [20] and human stool samples [21]. This significant discrepancy may be related to low concentration of the cells [22], presence of dead cells [23], presence of competitive microflora [24], injured cells and cells in a viable but non-culturable (VBNC) state [25], cell-free DNA [26], or may also be due to the presence of free Stx phage particles in the samples [27,28], resulting in the PCR-positive/culture-negative phenomenon [29,30,31,32]. The investigation of these occurrences and their underlying causes will support risk assessment and method enhancement for STEC detection. In this study, three different protocols in addition to the standard protocol were applied to test samples to assess the cause of *stx* PCR-positive culture-negative samples identified by a standard real time quantitative PCR protocol in ovine recto-anal junction samples. The generated data can be seen as representative of other matrices and is a further step towards an improved understanding of the STEC PCR-positive/culture-negative phenomenon.

## 2. Materials and Methods

### 2.1. Sheep Recto-Anal Junction Samples Examined for STEC

**Figure 1:**
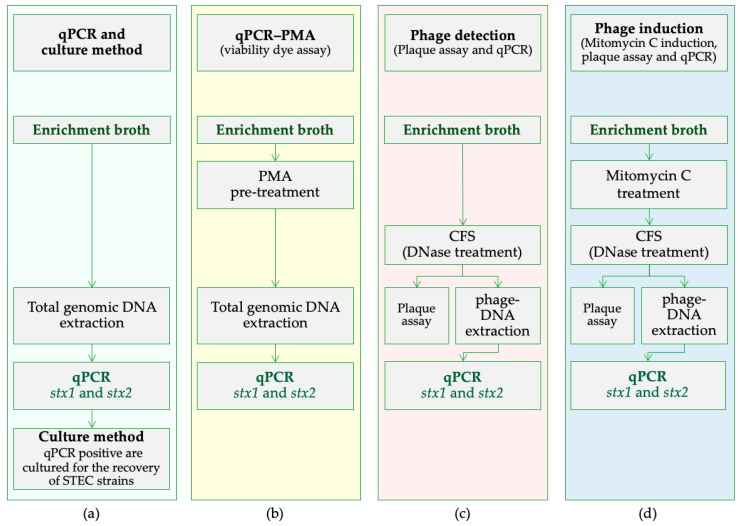
Methods used to investigate the cause of PCR-positive/culture-negative samples for STEC. The methods were applied to the examination of sheep recto-anal junction swab samples. The methods were as follows: (**a**) real-time PCR (qPCR) and culture method; (**b**) propidium monoazide pre-treatment and real-time PCR (qPCR-PMA); (**c**) bacteriophage detection and plaque assay; (**d**) phage induction. qPCR = real-time PCR; CFS = cell free supernatant; PMA = propidium monoazide.

In this study, 155 ovine recto-anal junction (o-RAJ) swab samples were collected between February and May 2019 from two different ovine slaughtering facilities. As this study utilised recto-anal junction swab samples which were collected from sheep in the abattoir after slaughter it was not necessary to obtain approval from the animal ethics committee. One foam-over-cotton head wood handle swab (VWR, Dublin, Ireland) pre-soaked in 1 mL of maximum recovery diluent (Oxoid, Dublin, Ireland) was inserted approximately 3–5 cm into the RAJ area using a rapid in and out motion. The samples were transported to the laboratory and stored at 4 °C, and microbiological analyses were started within 24 h after sampling. Each swab was transferred into 30 mL of modified Tryptone Soya Broth (TSB, Oxoid, Dublin, Ireland) with 16 mg/l novobiocin (Oxoid, Basingstoke, UK) and incubated at 41.5 °C for 24 h obtaining the enriched samples. The four protocols used in this study are presented in Figure 1 and are as follows: (a) standard qPCR and culture approach; (b) viability dye assay to identify the presence of dead cells; (c) to assess for presence free phage and; d) phage induction. Method (a) was used on all samples and methods (b), (c) and (d) on PCR-positive, culture-negative samples.

### 2.2. Real-Time PCR (qPCR) and Culture

Following enrichment, the total genomic DNA was extracted with InstaGene Matrix (Bio-Rad, Watford, UK). Briefly, 1 mL of each enriched sample was centrifuged for 10 min at 10,000× *g*, the pellet was resuspended in 1 mL of phosphate-buffered saline (PBS) (Oxoid, Basingstoke, UK), then centrifuged at 10,000× *g* for 10 min. The obtained pellet was resuspended in 100 µL of matrix and incubated at 56 °C for 30 min followed by incubation at 95 °C for 15 min. The suspensions were centrifuged at 15,000 × *g* for 5 min and supernatants were used as real-time PCR templates in a duplex real-time PCR assay for the detection of the genes *stx1* and *stx2* and performed on the LightCycler 480 (LC480) (Roche Diagnostics, Burgess Hill, UK). The primers and hydrolysis probes used are reported in Table 1.

Each reaction consisted of 2.4 μL of 5× LightCycler Multiplex Master (Roche Diagnostics, Burgess Hill, UK), 0.1 μL hydrolysis probes (0.1 μM), 0.7 μL of the forward and reverse primers (0.6 μM), and 2 μL of target template DNA, made up a 12 μL volume reaction with water. A positive and negative DNA control and a no template control (NTC) were included in each real-time PCR run. The cycling conditions were as follows: pre-incubation at 95 °C for 5 min, followed by 42 cycles (95 °C for 15 s, 60 °C for 60 s and 72 °C for 1 s). Data analysis was performed using the real-time Lightcycler PCR software. Positives samples were then plated on MacConkey agar (Oxoid, Basingstoke, UK) and incubated at 37 °C for 24 h.

A total of five pools containing five single colonies for each sample were extracted, suspending the colonies in 100 µL of PBS and proceeding with DNA extraction. The five single colonies were then collected and characterized from the positive pools. The positive control strains included *E.coli* O157:H7 (ATCC 35150) and *E.coli* O26:H11 (CDC 03-3014) (both *stx1* and *stx2* positive) and were cultured in TSB at 37 °C for 18 h, harvested by centrifugation (10 min at 10,000× *g*) and washed with PBS. Cells were then re-suspended in 100 µL of InstaGene matrix and DNA extracted following the described protocol.

### 2.3. Pre-Treatment with Propidium Monoazide and Real Time PCR (qPCR-PMA)

In order to investigate the presence of dead cells (Figure 1b), the enriched samples were treated with a 25 µM final concentration of propidium monoazide—PMAxx (Biotium, Fremont, CA, USA) and stored at room temperature in the dark for 10 min. The samples were exposed to intense visible light for 20 min with the use of the LED Photolysis Device (PMA-Lite, Biotium, Fremont, CA, USA). The treated cultures were centrifuged at 5000× *g* for 10 min and the pellets were used as templates for total genomic DNA extraction. In addition, 500 µL of the positive control strains were inactivated at 95 °C for 5 min (heat killed controls, HK) and used as dead cells suspension controls. 250 µL of the HK positive controls were resuspended with 250 µL of corresponding live positive controls to generate a 50% (*v*/*v*) mixture of live and dead cells suspension from which DNA was extracted following the PMA treatment protocol.

### 2.4. Detection of Free Bacteriophages

To investigate if there were free bacteriophages in the samples (Figure 1c), samples were purified by centrifuging 2 mL of enriched samples and the supernatants were passed through low-protein-binding 0.22 µm-pore-size membrane filters (Agilent Technology, Didcot, UK), obtaining the cell free supernatants (CFS). One ml of CFS was plated on tryptone soya agar (TSA) (Oxoid, Dublin, Ireland) and incubated at 37 °C for 24 h in order to verify the absence of bacteria. The putative bacteriophage suspensions were then treated with DNase (100 units/mL of CFS) (Sigma-Aldrich, Wicklow, Ireland) to eliminate free DNA outside the phage particles, and incubated at 37 °C for 1 h, after which 10 µL of EDTA (Sigma-Aldrich, Wicklow, Ireland) (final concentration 0.5 mM) was added to inactivate the DNase and incubated at 65 °C for 10 min (purified phage suspension, PPS). Two aliquots of the samples at this stage were used for the plaque assay (aliquot 1) described below and as a template for *stx1* and *stx2* qPCR (aliquot 2) in order to confirm the absence of free target DNA. The DNA from all phages, presumably present in the PPS, was isolated with a digestion treatment, adding 5 µl of Proteinase K (Sigma-Aldrich, Wicklow, Ireland) per ml of PPS and incubated at 56 °C for 1h. This final bacteriophage DNA suspension was purified with a phenol-chloroform (1:1, *v*/*v*) treatment, as described previously [34].

### 2.5. Phage Induction

To determine the presence of inducible phage among the samples, one aliquot of 5 mL of the enriched sample was transferred to a fresh tube containing mitomycin C (MMC) to a final concentration of 0.5 μg/mL (Figure 1d). The samples were incubated for 18 h at 37 °C in the dark on a shaker. The induced-phage DNA was prepared following the protocol described for the phage detection.

### 2.6. Plaque Assay

The purified phage suspensions obtained from the enriched sample and from the MMC pre-treated samples were diluted 10-fold and tested for plaque production. Briefly, 0.5 mL of an overnight Luria-Bertani (LB) broth (Oxoid, Dublin, Ireland) culture of the indicator *E.coli* K12 strain C600 containing 2 × 10^8^ CFU/mL was added to 100 μL of 0.1 M CaCl_2_ and incubated for 30 min at 37 °C. The suspensions were then gently mixed with 5 mL of soft-TSA (tryptone soya broth with 0.75% *v*/*v* agar), poured onto TSA plates and allowed to dry; 10 μL of each phage suspension and the 10-fold dilutions were poured on the plates creating a spot onto the bacterial lawn. After incubation for 18 to 24 h at 37 °C, the plates were examined for the presence of lysis zones indicating the presence of free phage particles in the sample. For infectivity studies and phages stocks, the plaques of lysed samples in qPCR-positive and culture-negative samples were removed with a wire loop and suspended in 500 μL of TSB. The suspensions were filtered through low-protein-binding 0.22-μm-pore-size membrane filters and 10 μL were spotted onto a monolayer of the host strains as described above for infectivity tests.

## 3. Results

Among the o-RAJ samples (*n* = 155) examined, method (a) (qPCR coupled with culture), showed 121 samples (78.1%) positive for STEC DNA, but it was only possible to isolate STEC from 68 of the samples (43.9%), i.e., a PCR-positive, culture-negative rate of 34.2% (53 samples). In total, 82 strains were recovered (>1 isolate from some samples) and differing *stx* profiles were observed. The majority of the strains were positive for both *stx* genes (*n* = 41), whilst 23 strains were positive for *stx1* only and 18 strains were positive for *stx2* only.

### 3.1. Real-Time PCR Results and Comparison with PMA-qPCR

The crossing point (Cp) values obtained with the qPCR were compared with the Cp values of the samples pre-treated with PMAxx. The effects of PMAxx-mediated inhibition of amplification of the control strain DNA is shown in Figure S1A,B for *stx1* and *stx2*, respectively (Supplementary Material), by using DNA derived from live cells (amplification curve 1), HK suspension (amplification curve 3) and a 50% (*v*/*v*) mixture (amplification curve 2). The results showed a 15-Cp value difference between the live cells and HK, demonstrating that PMAxx treatment efficiently suppressed DNA amplification from the dead cells for both targets.

For this study, the untreated and PMAxx-treated samples were analysed in the same qPCR runs in order to evaluate the effect of the PMA-mediated inhibition on the obtained Cp values. The samples were considered negative for the targeted sequences if the Cp values were greater than 30 and if the Cp from PMA-qPCR was greater than the value (cutoff = 2 Cp) of the non-treated samples (Cp PMA-qPCR>Cp qPCR), this signal was deemed to be from mostly dead cells. Among the PCR-positive/culture-negative samples (*n* = 53), the comparison of the standard qPCR with the PMA-qPCR highlighted 31 samples in total (59.6% of 53 samples) with amplification from dead or damaged cells in culture.

### 3.2. Detection of Free Bacteriophages and Inducible Prophages

In this study, all PCR-positive culture-negative samples were tested for the presence of free bacteriophage particles in the enrichment and the presence of MMC-inducible prophages through a plaque assay. Among the 53 samples, 17 (11.0%) were positive for the plaque assay, but only four (2.6%) samples were positive with the CFS-qPCR. In particular, one CFS sample was positive for *stx1* and three CFS samples were positive for *stx2* (Table 2). In total, 26 samples pre-treated with MMC were positive for the formation of plaques (16.8%). Among those, 13 samples were positive with the CFS-qPCR for at least one *stx* gene, including six CFS samples positive for *stx2* and seven CFS samples positive for both *stx1* and *stx2.*

In 13 CFS samples (8.4%) and 13 MMC pre-treated CFS samples (8.4%), which were positive for the plaque assay, it was not possible to detect the *stx* genes by qPCR.

Sample 12 was positive for Stx1 phage DNA and samples 51, 55 and 99 were positive for Stx2 phages and were also positive for the plaque assay. These enriched samples were positive at the qPCR for both *stx* genes, except Sample 99 which was positive for *stx1* only (Table 3). Aliquot 2, collected for confirming the absence of free target DNA in the PPS, resulted negative to *stx1* and *stx2* qPCR.

## 4. Discussion

Different studies have reported the detection of STEC by PCR or qPCR from different matrices [35,36,37,38] and the challenges of subsequently obtaining cultured STEC isolates. This significant discrepancy may be related to dead and injured cells, cells in a viable but non-culturable (VBNC) state, the presence of free DNA and free Stx bacteriophages in the samples [10,39,40,41,42,43,44]. In addition, the microbial communities can have an important influence on the survival capabilities of STEC in environmental substrates and consequential cultural isolation [24,45]. 

In this study we applied a number of different methods to better understand the phenomenon and evaluate their application in analysing STEC results. In this case the methodology was applied to o-RAJ swab samples, but the approach should be applicable to other sample types also. With this approach, the percentage of samples which were PCR-positive/culture-negative without an explanation were reduced from 53 to 20 samples (34.2% to 12.9% of 155 samples, respectively), demonstrating that there is a need to develop novel approaches to better understand this phenomenon. A total of five pools containing five single colonies for each sample were picked-up from the agar and extracted, and from positive pools, the five single colonies were collected and characterized. The number and diversity of the agar reflects the variety of fermentative profiles of different STEC, which would suggest the use of several agars, but the percentage obtained of recovered culturable STEC in this study (PCR-positive/culture-negative samples), were similar to other reports [19,20,21,29,36].

The PMA-qPCR provided valuable information to asses if viable cells were present in the sample, supporting risk management. In addition, the evaluation of the presence of free bacteriophages demonstrates the complexity of the samples and that the incidence of culture-negative samples is often due to different agents and conditions within the ecology of the sample.

**Figure 2:**
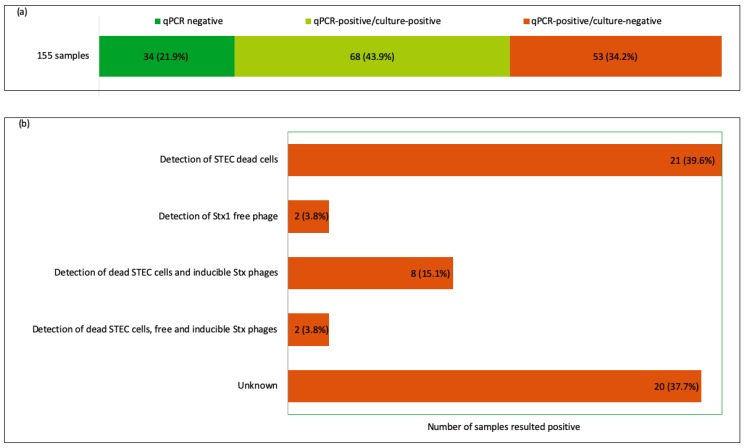
Results of the approaches used in this study. (**a**) Results of the STEC screening (method a) of the o-RAJ samples (*n* = 155). (**b**) Results of the combination of the methods used to investigate the qPCR-positive/culture-negative phenomenon (*n* = 53).

The combination of the results with the PMA-qPCR demonstrated the presence of dead cells in samples which tested PCR-positive by the standard assay. The presence of dead or damaged cells in culture was detected in 31 samples (58.5% of the PCR-positive/culture-negative), which were not able to form colonies in culture, illustrating why cultural isolation was not successful and demonstrating their contribution to the PCR-positive, culture-negative phenomenon. In this study, it was possible to detect two samples with free Stx phage particles (3.8% of PCR-positive/culture-negative samples) in CFS but with the use of PMA-qPCR it was also possible to identify dead cells in the samples, together with free Stx particles and inducible Stx phages in two samples (3.8%) and in eight samples with inducible Stx phages (15.1%) (Figure 2b), further demonstrating the differing causes for culture negative samples.

The *stx1* and *stx2* genes are mobile virulence factors, located on the genome of temperate bacteriophages [46,47,48,49] which are able to integrate in the *E.coli* genome during lysogeny, resulting in acquired ability to express the Shiga toxin [29]; however, the prophages can be induced to enter the lytic cycle producing toxin and new phage particles [50]. In this study, mitomycin C was used for the lytic cycle induction in the enriched samples resulting in an increase of number of positive samples for the plaque assay (Table 2, 35 samples). The same samples were tested for the extraction of free phage particles and 20 o-RAJ samples were positive for the amplification of at least one *stx* gene, suggesting the presence in the enriched samples of STEC integrated prophages, even though the cells were not culturable. Ten samples were plaque positive after the MMC pre-treatment even though were negative for free bacteriophages with method (c) (Figure 1); seven of them were in fact negative for both the *stx* genes and three were positive for *stx2.* Interestingly, Sample 63 was negative for free phage particles in culture (CFS) but was positive for both *stx1* and *stx2* after induction. This result may be due to the presence of low number of STEC cells in the samples with inducible prophages; in fact, metabolically active cells, rather than dormant cells, are required by bacteriophages to replicate their nucleic acids. It was not possible to confirm that the phages from the positive plaques were infective. This may be due to the possibility that the phages, instead of replicating in the host strains causing lysis, remained integrated in the bacterial genome and generated lysogens as suggested by Imamovic and Muniesa [51]. In this study, TSB modified with 16 mg/L novobiocin (mTSB) was used as enrichment broth for 24h of incubation. It has been reported that for some serogroups and concentrations higher than 20 mg/L the growth of such strains is slowed down or has an effect on the viability of STEC [52]. However, the standard method ISO/TS 13136:2012 indicates 16 mg/L as a concentration of novobiocin in the mTSB for the enrichment step, which was used in different studies [19,53,54,55,56].

The deficit between PCR- and culture-positive prevalence rates is comparable with other reported surveys [57,58] and with the combination of the methods used in this study it was possible to evaluate the effect of Stx bacteriophages and dead cells on the positivity of qPCR. Sample 12 was positive for *stx1* and *stx2* with the standard qPCR (enrichment sample) and free *stx1* phage was detected but the samples resulted negative for the induction, indicating the presence of only free *stx1* bacteriophage particles in the sample (Table 3). Samples 51 and 55 were positive for the amplification of the *stx1* and *stx2* genes in the enriched samples, and free *stx2* phage particles were also detected. Additionally, induced prophages were detected in both sample 51 (*stx1*/*stx2* detected) and only the *stx2* in sample 55 (Table 3). Interestingly, Sample 99 tested positive for *stx1* in the enriched sample (Table 3) but only *stx2* phage DNA was detected in the CFS and both *stx* genes were detected in the induced samples. This result suggested the presence of free *stx2* phage in the sample and also the presence of *stx1* and *stx2* induced prophage in cells. The number of cells with *stx2* gene was probably present in the sample in a low number, not detectable with the qPCR.

Two samples contained free *stx*-encoding phages and, in total, 31 samples with dead cells were detected, with or without free phage particles and inducible prophages. In total, 13 samples were plaque positive but the qPCR for the *stx* genes resulted negative. This result demonstrates the complexity of the samples and the presence of a heterogeneous population of bacteriophages and prophages in the microbiota of the tested animals unrelated to *stx* carriage which might also cause the formation of the plaques.

Whilst it was not possible to explain the qPCR-positive culture-negative phenomenon for 20 samples, the generated data demonstrate the reduction of the number of the occurrences of the phenomenon from 34.2% to 12.9% and represents a further step towards improving the understanding of the biology of this phenomenon, giving a valid support for risk managers and public health prospective regarding qPCR-positive/culture-negative results for o-RAJ swab samples which requires a public health risk assessment.

## Figures and Tables

**Table 1:** Primers and probes used for the amplification of *stx* genes.

Gene	Description	Primer Name	Amplicon Size (bp)	References
*stx1*	Forward	TTTGTYACTGTSACAGCWGAAGCYTTACG	131	[33]
	Reverse	CCCCAGTTCARWGTRAGRTCMACRTC		
	Probe	FAM-CTGGATGATCTCAGTGGGCGTTCTTATGTAA-BHQ1		
*stx2*	Forward	TTTGTYACTGTSACAGCWGAAGCYTTACG	128	[33]
	Reverse	CCCCAGTTCARWGTRAGRTCMACRTC		
	Probe	Hex- TCGTCAGGCACTGTCTGAAACTGCTCC-BHQ2		

**Table 2:** *Stx* gene profiles, tested by qPCR, of the samples positive with the plaque assay. These include samples examined for free phage (Figure 1c) and for phage induction (Figure 1d).

Sample *n*	qPCR/Culture Result	Detection of Free Bacteriophages			MMC Treatment		
		*Stx1*	*Stx2*	Plaque	*Stx1*	*Stx2*	Plaque
12	pos/neg	+	−	+	−	−	+
31	pos/neg	−	−	+	−	−	+
32	pos/neg	−	−	−	−	−	+
51	pos/neg	−	+	+	+	+	+
52	pos/neg	−	−	+	−	−	+
53	pos/neg	−	−	+	−	+	+
54	pos/neg	−	−	+	+	+	+
55	pos/neg	−	+	+	−	+	+
57	pos/neg	−	−	+	−	−	+
60	pos/neg	−	−	−	−	−	+
62	pos/neg	−	−	+	−	−	+
63	pos/neg	−	−	−	+	+	+
74	pos/neg	−	+	−	−	+	+
75	pos/neg	−	+	−	+	+	+
76	pos/neg	−	−	+	−	+	+
77	pos/neg	−	−	−	−	+	+
88	pos/neg	−	−	+	−	−	+
92	pos/neg	−	−	−	−	−	+
93	pos/neg	−	−	+	+	+	+
94	pos/neg	−	−	+	+	+	+
97	pos/neg	−	−	−	−	−	+
99	pos/neg	−	+	+	+	+	+
109	pos/neg	−	+	−	−	−	+
125	pos/neg	−	−	+	−	−	+
151	pos/neg	−	−	+	−	−	+
152	pos/neg	−	−	+	−	+	+

qPCR: real-time PCR; pos: positive, neg: negative; MMC = Mitomycin C.

**Table 3:** Effect of the *stx* bacteriophages to qPCR-positive/culture-negative results.

Sample *n*	Enriched Samples		Detection of Free Bacteriophages			MMC Treatment		
	*Stx1*	*Stx2*	*Stx1*	*Stx2*	Plaque	*Stx1*	*Stx2*	Plaque
12	+	+	+	−	+	−	−	−
51	+	+	−	+	+	+	+	+
55	+	+	−	+	+	−	+	+
99	+	−	−	+	+	+	+	+

MMC = Mitomycin C.

## Supplementary Materials

The following are available online at https://www.mdpi.com/2076-2607/8/4/587/s1, Figure S1: PMAxx qPCR amplification curves of the gene *stx1* (**A**) and *sxt2* (**B**) of the *E.coli* O157:H7 (ATCC35150).
