# Supplementary material for: Investigation of the Causes of Shigatoxigenic Escherichia coli PCR Positive and Culture Negative Samples
Source: Microorganisms. 2020 Apr 18;8(4):587. doi: 10.3390/microorganisms8040587 (PMC7232186; doi:10.3390/microorganisms8040587)
Supplement: Supplementary file 1 [file microorganisms-08-00587-s001.pdf]

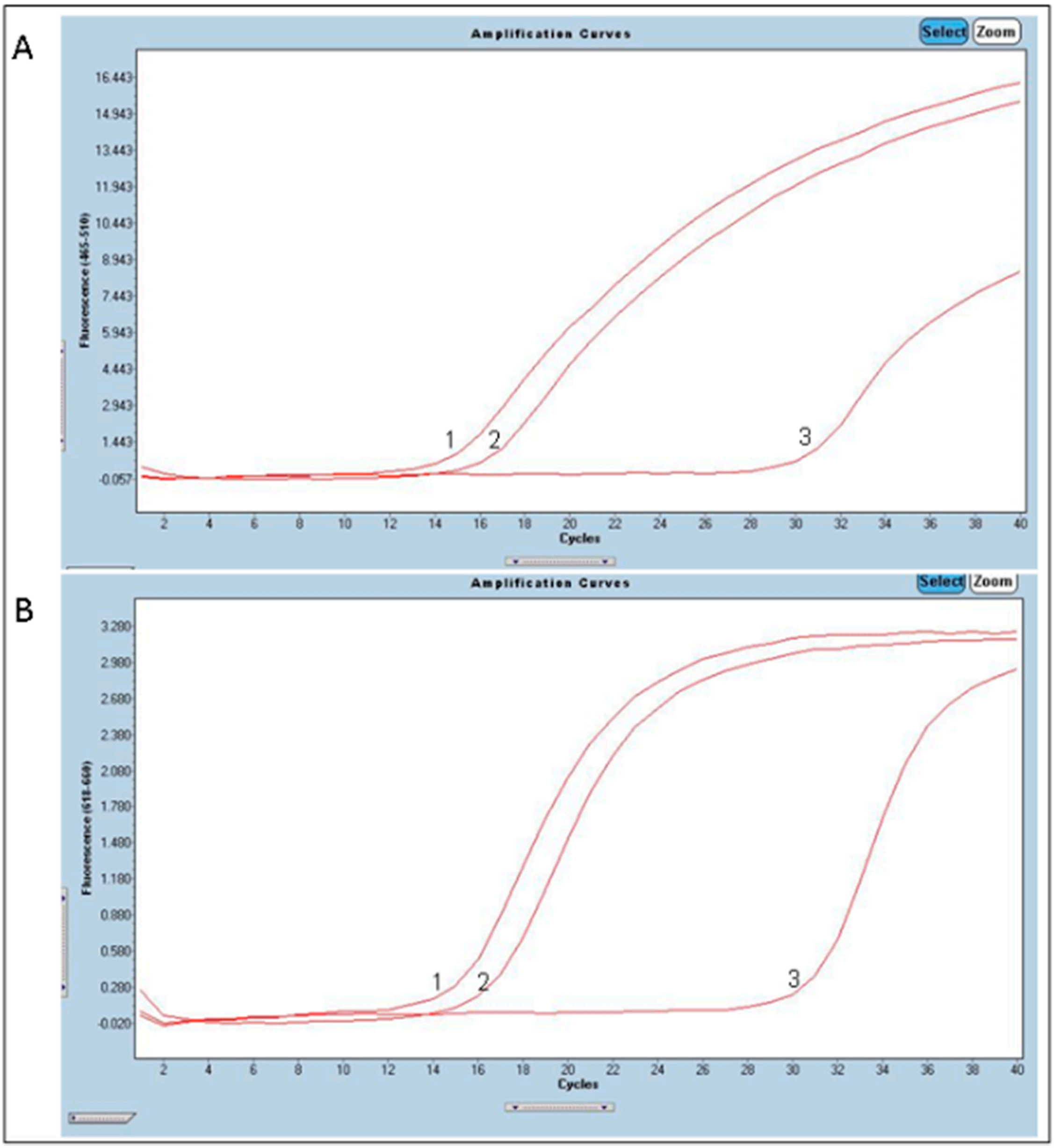

Figure S1. The effects of PMAxx-mediated inhibition of amplification of the control strain DNA for *stx1* and *stx2*, respectively
